# Zero-Watermarking Algorithm for Medical Image Based on VGG19 Deep Convolution Neural Network

**DOI:** 10.1155/2021/5551520

**Published:** 2021-07-01

**Authors:** Baoru Han, Jinglong Du, Yuanyuan Jia, Huazheng Zhu

**Affiliations:** ^1^College of Medical Informatics, Chongqing Medical University, Chongqing, China; ^2^College of Intelligent Technology and Engineering, Chongqing University of Science and Technology, Chongqing, China

## Abstract

Aiming at the security issues in the storage and transmission of medical images in the medical information system, combined with the special requirements of medical images for the protection of lesion areas, this paper proposes a robust zero-watermarking algorithm for medical images' security based on VGG19. First, the pretrained VGG19 is used to extract deep feature maps of medical images, which are fused into the feature image. Second, the feature image is transformed by Fourier transform, and low-frequency coefficients of the Fourier transform are selected to construct the feature matrix of the medical image. Then, based on the low-frequency part of the feature matrix of the medical image, the mean-perceptual hashing algorithm is used to achieve a set of 64-bit binary perceptual hashing values, which can effectively resist local nonlinear geometric attacks. Finally, the algorithm adopts a watermarking image after scrambling and the 64-bit binary perceptual hashing value to obtain robust zero-watermarking. At the same time, the proposed algorithm utilizes Hermite chaotic neural network to scramble the watermarking image for secondary protection, which enhances the security of the algorithm. Compared with the existing related works, the proposed algorithm is simple to implement and can effectively resist local nonlinear geometric attacks, with good robustness, security, and invisibility.

## 1. Introduction

With the construction of hospital normalization, digitization is widely applied in the medical field [1]. Modern medical equipment produces lots of digital medical images every day [2, 3]. Owing to the rapid development of information technology, intelligent medicine and remote diagnosis are becoming more and more mature [4–6]. A large number of medical images are often transmitted through the Internet [7]. Unauthorized persons can easily obtain, store, use, and tamper with medical images on the network [8]. In this scene, sensitive information, such as patient information recorded in medical images, is also easy to leak. With the development of the medical information system, medical image sharing and remote diagnosis technology are becoming more mature [9]. In the application of these techniques, it becomes more and more urgent to protect patient's information, such as personal information in magnetic resonance imaging (MRI) and other medical images, as well as electronic medical record data from being leaked [10, 11]. Therefore, the problem of medical information security has become an urgent problem.

Medical image watermarking can effectively solve the above problem and provide safe and reliable protection for medical information. Current information security of the medical information system relies on the development of modern information technology [12]. Traditional encryption methods of cryptography have great limitations and high risks. It is unable to identify the copyright of data at the technical level, and it has been unable to adapt to the changing information environment. Digital watermarking is a kind of information security technology, which can be applied to image integrity authentication and image copyright protection [13, 14]. Originally, digital watermarking was used for copyright protection of digital multimedia on the Internet. With the increasing demand for information security in the medical field, the invisibility and robustness of digital watermarking are used to hide patient's personal information in the medical image to secure transmission on the Internet. By means of digital watermarking, specific identification information is embedded into carrier images, so that the authenticity and integrity authentication of medical images, Electronic Patient Record (EPR) hiding, and copyright protection can be realized. Therefore, medical image watermarking has an extremely important application value, which attracts continuous attention from researchers [15, 16].

Medical image watermarking generally chooses different watermarking algorithms according to invisibility, robustness, and embedding capacity. The invisibility, robustness, and capacity of watermarking restrict each other, but most of the current medical image watermarking algorithms only focus on one of them. Medical images are mostly single channel gray images, and the details contained in the pixels are very important. Any changes may cause distortion, which will affect doctor's diagnosis. However, the application of traditional image watermarking to medical images can easily cause the distortion of medical images, which may lead to potential misdiagnosis. In order to reduce the influence of watermarking on the original information of the medical image, it is very important to design a medical image watermarking algorithm which is lossless to the original image. Since the robust zero-watermarking adopts the way of zero-embedding, the original medical image is not modified. While realizing protection, the robust zero-watermarking takes into account the robustness and capacity of the watermarking and will not destroy the integrity of the medical image. Therefore, it is very suitable for the medical image [17–19].

Robust medical image zero-watermarking mainly focuses on the ability of the algorithm to resist various attacks, that is, the algorithm can completely extract the zero-watermarking information after various attacks [20–22]. The geometric attack will change the position relationship between the attacked medical image and the original medical image, resulting in serious out of synchronization between zero-watermarking information and medical images, which makes it is extremely difficult to extract zero-watermarking. Therefore, the ability of the algorithm to resist geometric attacks has become a research hotspot in the field of medical image zero-watermarking [1, 12]. But these geometric attacks mainly refer to global geometric transformation (rotation, scaling, translation, etc.), which is a kind of linear transformation. However, in practical applications, there is usually another type of geometric attack, which belongs to the local nonlinear geometric attack. At present, there are a few watermarking algorithms that can resist local nonlinear geometric attacks, which make almost all watermarking algorithms invalid. Compared with the global geometric attack, the local nonlinear geometric attack is more difficult to implement.

To solve this problem, a zero-watermarking algorithm based on VGG19 against local nonlinear geometric attacks is proposed in this paper. In this algorithm, VGG19 can extract complex high-level features using a deep structure with powerful representation and learning ability. At the same time, the network structure has good geometric invariance and can extract image features with high and good robustness. The algorithm uses a perceptual hashing algorithm based on deep features to construct zero-watermarking, which has good robustness against local nonlinear geometric attacks. Hermite chaotic neural network is used to scramble the watermarking image for secondary protection, and it can enhance the security of the algorithm. Experimental results show that the proposed algorithm can effectively resist local nonlinear geometric attacks and display the superior performance of the proposed algorithm compared with other watermarking algorithms.

## 2. The Fundamental Theory

### 2.1. Feature Extraction Based on VGGnet

**Figure 1:**
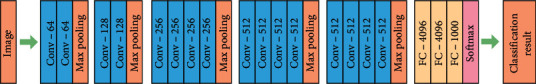
The structure of the VGG19 network.

VGGnet is a representative type of deep convolutional neural network (CNN), which is often used in feature extraction and transfer learning [23]. The most widely used VGGnet is VGG19, which consists of 19 hidden layers (16 convolution layers and 3 fully connected layers), as shown in Figure 1. VGG19 uses a series of 3 × 3 convolution kernels to extract image features and expands the number of feature channels through convolution layers. Let *W*_*i*_ and *b*_*i*_ represent the weights and bias of the *i*th convolution layer, the feature can be extracted by

(1) $$\begin{array}{c} X_{i}^{\text{out}}=\sigma \left(W_{i}*X_{i}^{\text{in}}+b_{i}\right), \end{array}$$

where *X*_*i*_^out^ and *X*_*i*_^in^, respectively, denote the input and output feature maps and *σ*(·) is the rectified linear unit (ReLU). In each convolution layer, the stride is set to 1. To avoid the explosion of calculation, VGG19 uses max pooling layers to reduce the size of the feature maps.

In fully connected layers, each node of the given layer is connected with all nodes of its previous layers, which can map the distributed feature representation to the sample label space by

(2) $$\begin{array}{c} Y={\text{FC}}_{3}\left({\text{FC}}_{2}\left({\text{FC}}_{1}\left(P\left(X_{16}^{\text{out}}\right)\right)\right)\right), \end{array}$$

where FC(·) denotes the operation of the fully connected layer and *P*(·) refers to the max pooling operation.

At the end of VGG19, a softmax layer produces the classification result of the image:

(3) $$\begin{array}{c} Y_{j}=\frac{e^{z_{j}}}{\sum_{c=1}^{C}e^{z_{c}}}, \end{array}$$

where *Y*_*j*_ is the probability of the *j*th node and *z*_*j*_ and *C*, respectively, denote the output of *j*th node and the number of the classification.

Compared with other types of CNNs, VGG19 improves the depth of the network and adopts the alternate structure of many convolution layers and nonlinear activation layers, which is beneficial to extract accurate features. In this work, different from image classification tasks, we just use the convolution layers and max pooling layers from the pretrained VGG19 as the preprocessing method to extract deep feature maps from medical images. Unlike other medical zero-watermarking algorithms, our method can extract abstract high-level features from medical images to improve the antigeometric attack ability of zero-watermarking.

### 2.2. Discrete Fourier Transform

Discrete Fourier transform plays an important role in the development of signal analysis and processing. Because of its definite physical meaning, discrete Fourier transform is widely used in many fields of signal analysis and processing.

#### 2.2.1. One-Dimensional Discrete Fourier Transform

Let *f*(*x*) denote a time domain function of *x*, where *x* represents a time domain variable and *u* is a frequency domain variable. When *f*(*x*) reaches the Dirichlet condition, the discrete Fourier transform is formula (4) and the inverse transform is formula (5), where *F*(*u*) is a frequency domain function:

(4) $$\begin{array}{c} F\left(u\right)=\sum_{x=0}^{N-1}f\left(x\right)e^{-\left(j2\pi ux/N\right)},\;u=0,1,\ldots ,N-1, \end{array}$$

(5) $$\begin{array}{c} f\left(x\right)=\frac{1}{N}\sum_{u=0}^{N-1}F\left(u\right)e^{\left(j2\pi ux/N\right)},\;x=0,1,\ldots ,N-1. \end{array}$$

#### 2.2.2. Two-Dimensional Discrete Fourier Transform

For a given image with the size of *M* × *N*, when it satisfies the Dirichlet condition, the two-dimensional discrete Fourier positive transform is

(6) $$\begin{array}{c} F\left(u,v\right)=\sum_{x=0}^{M-1}\sum_{y=0}^{N-1}f\left(x,y\right)e^{-j\left(2\pi /M\right)xu}e^{-j\left(2\pi /N\right)yv},\;u=0,1,\ldots ,M-1;v=0,1,\ldots ,N-1. \end{array}$$

Its inverse transformation formula is

(7) $$\begin{array}{c} f\left(x,y\right)=\frac{1}{MN}\sum_{u=0}^{M-1}\sum_{v=0}^{N-1}F\left(u,v\right)e^{j\left(2\pi /M\right)xu}e^{j\left(2\pi /N\right)yv},\;x=0,1,\ldots ,M-1;y=0,1,\ldots ,N-1, \end{array}$$

where *x* and *y* are the values in the space domain and *u* and *v* are the values in the frequency domain.

**Figure 2:**
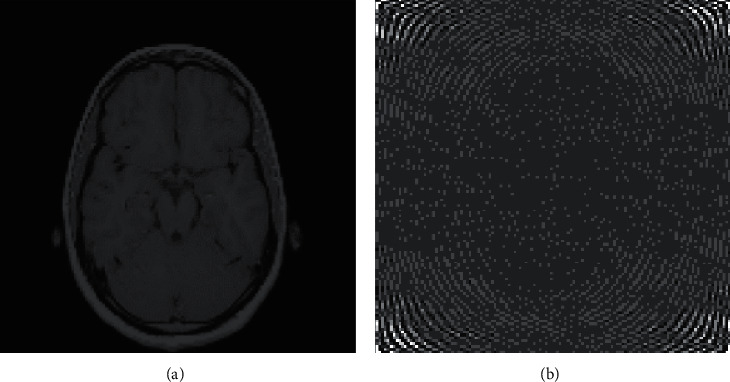
Discrete Fourier transform of the medical image. (a) Original medical image. (b) Medical images in the Fourier domain.

Two-dimensional discrete Fourier transform transforms an image from the space domain to the frequency domain, which has a clear physical meaning. The frequency of an image is an index that characterizes the intensity of the gray level change in the image and is the gradient of the gray level in the plane space. After the Fourier transform, the changes in the flat area of the image are represented by low-frequency coefficients, and the details of the image are represented by high-frequency coefficients.  Figure 2 shows the result of the Fourier transform of a medical image.

After the two-dimensional discrete Fourier transform, the transform coefficient matrix of the image shows that if the origin of the transform matrix is set at the center, the spectral energy is concentrated near the center of the short transform coefficient matrix. If the origin of the two-dimensional discrete Fourier transform matrix is set in the upper left corner, the energy of the image will be concentrated in the four corners of the coefficient matrix. This is determined by the nature of the two-dimensional discrete Fourier transform itself. It also shows that the image energy is generally concentrated in the low-frequency area. This determines that the two-dimensional discrete Fourier transform is very suitable for image processing. In this paper, a two-dimensional discrete Fourier transform is used to transform the features of the medical image into the Fourier domain to construct zero-watermarking.

### 2.3. Perceptual Hashing Algorithm

Perceptual hashing is considered as a one-way mapping from the multimedia data set to the perceptual content hashing value; that is, a short digital digest uniquely represents multimedia data with the same perceptual content. This digital digest is called perceptual hashing value, so the mapping process is also called the process of hash value generation.

Image perceptual hashing is usually called image digital fingerprint or image digital digest. It can map the image to a group of hash sequences, which greatly reduces the storage of the digital image and brings great convenience to image management and maintenance. It has become a research hotspot in the field of multimedia signal processing and security. Perceptual feature extraction is the core of the perceptual hashing algorithm. The effectiveness and reliability of perceptual feature extraction will directly affect the uniqueness and robustness of the perceptual hashing sequence.

**Figure 3:**
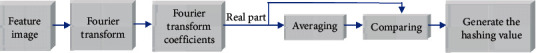
The flowchart of mean-perceptual hashing.

A mean-perceptual hashing algorithm is proposed in this paper. The mean-perceptual hashing algorithm uses the mean value of the elements of the medical image feature matrix to generate a hashing sequence. The construction of the feature image is realized by VGG19 and image fusion.  Figure 3 describes the flowchart of the mean-perceptual hashing algorithm.

### 2.4. Hermite Chaotic Neural Network

**Figure 4:**
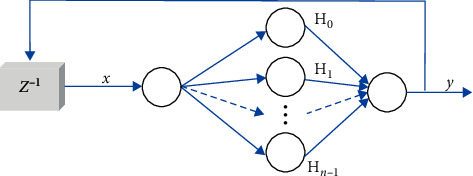
The architecture of Hermite chaotic neural network.

In this work, a new Hermite chaotic neural network is used to scramble the watermarking image [24]. The structure of the network is shown in Figure 4, whose topology is *m* × *n* × 1.

The hidden layer neuron input is

(8) $$\begin{array}{c} O_{j}=w_{j}x,\;j=0,1,2,\ldots ,n-1, \end{array}$$

where *w*_*j*_ is the weight between the input layer and hidden layer and *c*_*j*_ is the weight between the hidden layer and output layer. The hidden layer neuron output is

(9) $$\begin{array}{c} H_{j}\left(O_{j}\right),\;j=0,1,2,\ldots ,n-1, \end{array}$$

where *H*_*j*_( ),  *j*=0,1,2,…, *n* − 1 represents Hermite orthogonal polynomial terms. The Hermite orthogonal polynomial formula is as follows:

(10) $$\begin{array}{c} \left\{\begin{array}{c} H_{0}\left(x\right)=1,\\ H_{1}\left(x\right)=2x,\\ H_{k+1}\left(x\right)=2xH_{k}\left(x\right)-2kH_{k-1}\left(x\right),\;k=1,2,\ldots ,x\in \left(-\infty ,\infty \right). \end{array}\right. \end{array}$$

The output of the Hermite chaotic neural network is

(11) $$\begin{array}{c} y=\sum_{j=0}^{n-1}c_{j}H_{j}\left(O_{j}\right), \end{array}$$

where (*T*_*t*_, *d*_*t*_), *t* = 1, 2,…, *l* is the training sample set and *l* is the number of training samples. Hermite chaotic neural network input is *T*_*t*_=(*x*_1*t*_, *x*_2*t*_,…, *x*_*mt*_), and its desired output is *d*_*t*_. The backpropagation (BP) algorithm is used to train the network.

The weights of the network are trained according to the following formula:

(12) $$\begin{array}{c} e_{t}=d_{t}-y_{t},\\ E=\frac{1}{2}\sum_{t=1}^{l}e_{t}^{2},\\ \Delta c_{j}=-\eta \frac{\partial E}{\partial c_{j}},\\ \Delta w_{ij}=-\eta \frac{\partial E}{\partial w_{ij}}, \end{array}$$

where *t*=1,2,…, *l*, *i*=1,2,…, *m*,  *j*=1,2,…, *n*.

The chaotic sequence produced by the logistic chaotic function is used as a sample set. The logistic chaotic function is as follows:

(13) $$\begin{array}{c} x\left(n+1\right)=\mu x\left(n\right)\left(1-x\left(n\right)\right), \end{array}$$

where 3.5699456 ≤ *u* ≤ 4, *x*∈ (0, 1).

**Figure 5:**
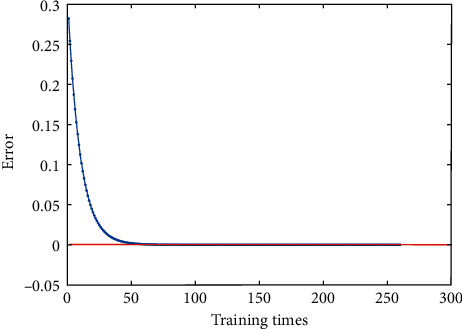
Training process.

**Figure 6:**
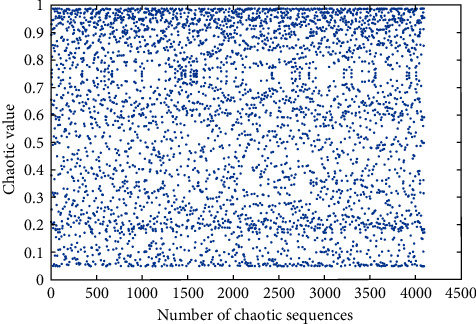
Chaotic sequence.

In this work, we set *μ*=3.8, *l*=1000, *E*=10^−12^, *η*=0.03, *n*=3, and the maximum number of training to 1500 epochs. The training process of the Hermite chaotic neural network is shown in Figure 5. When the number of training times is 260, the error is 9.5169*e*−13, which has reached the expected error. When the initial value of the Hermite chaotic neural network is 0.66, the chaotic sequence generated by the network is shown in Figure 6. The chaotic sequence is used to scramble the watermarking image. The number of the chaotic sequence is determined by the size of the watermarking image.

## 3. Zero-Watermarking Embedding and Extraction Algorithm

### 3.1. Embedding Algorithm

**Figure 7:**
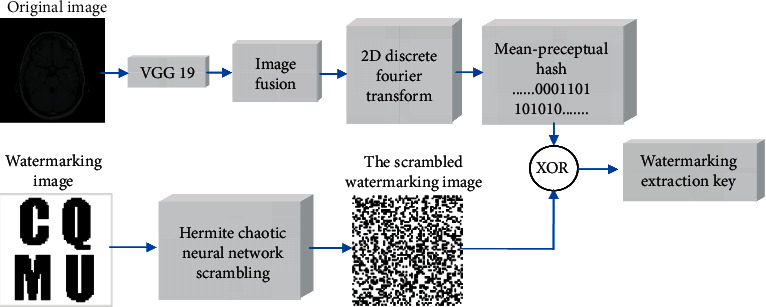
Watermarking embedding procedure.

Choosing an image with a specific meaning, our algorithm uses it as the original watermarking image in the experiment. It is recorded as *W*(*i*, *j*)={*w*(*i*, *j*)*|w*(*i*, *j*)=0,  1; 1 ≤ *i* ≤ *M*_1_, 1 ≤ *j* ≤ *M*_2_}. The gray value of the original watermarking image is represented as *w*(*i*, *j*). The original medical image is recorded as *F*(*i*, *j*)={*f*(*i*, *j*)*|f*(*i*, *j*) ∈ *R*,  1; 1 ≤ *i* ≤ *N*_1_, 1 ≤ *j* ≤ *N*_2_}. Here, the pixel gray value of the original medical image is represented by *f*(*i*, *j*). For the convenience of calculation, let *M*_1_=*M*_2_=64, *N*_1_=*N*_2_.  Figure 7 shows the watermarking embedding procedure.

(1) Hermite chaotic neural network is used to scramble the position of pixels in the original watermarking image *W*(*i*, *j*) to get the scrambled watermarking image *BW*(*i*, *j*).
(2) The pretrained VGG19 is used to extract the deep feature maps FM(*k*, *l*, *p*) of the original medical image *F*(*i*, *j*):
  (14) $$\begin{array}{c} F\left(i,j\right)⟶\text{VGG}19⟶\text{FM}\left(k,l,p\right), \end{array}$$
 where 1 ≤ *k* ≤ 8, 1 ≤ *l* ≤ 8, and 1 ≤ *p* ≤ 512.
(3) The deep feature maps FM(*k*, *l*, *p*) are fused to generate feature image FI(*k*, *l*):
  (15) $$\begin{array}{c} \text{FI}\left(k,l\right)=\sum_{p=1}^{512}\text{FM}\left(k,l,p\right). \end{array}$$
(4) Two-dimensional discrete Fourier transform is used to transform the feature image FI(*k*, *l*), and the transform coefficients FIF(*k*, *l*) are obtained to construct the feature matrix of medical image FIM(*k*, *l*):
  (16) $$\begin{array}{c} \text{FIF}\left(k,l\right)=2\;D-\text{DFT}\left(\text{FI}\left(k,l\right)\right),\\ \text{FIF}\left(k,l\right)⟶\text{FIM}\left(k,l\right). \end{array}$$
(5) The hashing sequence of medical image feature matrix FIM(*k*, *l*) is extracted by the mean-perceptual hashing algorithm to generate 64-bit binary hashing sequence *PH*(*q*):
  (17) $$\begin{array}{c} PH\left(q\right)=m\text{Hash}\left(\text{FIM}\left(k,l\right)\right), \end{array}$$
where 1 ≤ *q* ≤ 64.
(6) The 64-bit binary hashing sequence *PH*(*q*) is XORed with the scrambled watermarking image *BW*(*i*, *j*). And, the watermarking extraction key Key(*i*, *j*) is generated to extract the watermarking image:

(18) $$\begin{array}{c} \text{Key}\left(i,j\right)=PH\left(q\right)⊕BW\left(i,j\right), \end{array}$$

where 1 ≤ *q* ≤ 64, 1 ≤ *i* ≤ 64,  and 1 ≤ *j* ≤ 64. The watermarking extraction key Key(*i*, *j*) can be saved in a third party for later watermarking extraction.

### 3.2. Extraction Algorithm

**Figure 8:**
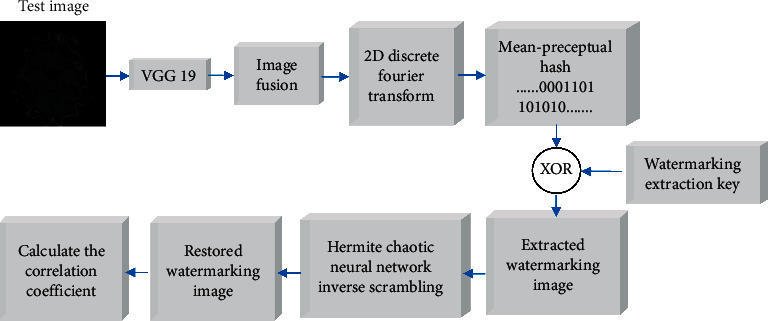
Watermarking extraction procedure.

The medical image to be tested is recorded as *F*′(*i*, *j*)={*f*′(*i*, *j*)*|f*′(*i*, *j*) ∈ *R*, 1;  1 ≤ *i* ≤ *N*_1_, 1 ≤ *j* ≤ *N*_2_}. The extraction algorithm is similar to the embedding algorithm, and the specific steps are as follows.  Figure 8 shows watermarking extraction procedure.

(1) The pretrained VGG19 is used to extract the deep feature maps FM′(*k*, *l*, *p*) of the medical image to be tested *F*′(*i*, *j*):
  (19) $$\begin{array}{c} F^{\prime }\left(i,j\right)⟶\text{VGG}19⟶\text{FM}{}^{\prime }\left(k,l,p\right), \end{array}$$
where 1 ≤ *k* ≤ 8,1 ≤ *l* ≤ 8,1 ≤ *p* ≤ 512.
(2) The deep feature maps FM′(*k*, *l*, *p*) are fused to generate feature image FI′(*k*, *l*):
  (20) $$\begin{array}{c} \text{FI}{}^{\prime }\left(k,l\right)=\sum_{p=1}^{512}\text{FM}{}^{\prime }\left(k,l,p\right). \end{array}$$
(3) Two-dimensional discrete Fourier transform is used to transform the feature image FI′(*k*, *l*), and the transform coefficients FIF′(*k*, *l*) are obtained to construct the feature matrix of medical image FIM′(*k*, *l*):
  (21) $$\begin{array}{c} \text{FIF}{}^{\prime }\left(k,l\right)=2\;D-\text{DFT}\left(\text{FI}{}^{\prime }\left(k,l\right)\right),\\ \text{FIF}{}^{\prime }\left(k,l\right)⟶\text{FIM}{}^{\prime }\left(k,l\right). \end{array}$$
(4) The hashing sequence of medical image feature matrix FIM′(*k*, *l*) is extracted by the mean-perceptual hashing algorithm to generate 64-bit binary hashing sequence *PH*′(*q*):
  (22) $$\begin{array}{c} PH^{\prime }\left(q\right)=m\text{Hash}\left(\text{FIM}{}^{\prime }\left(k,l\right)\right), \end{array}$$
where 1 ≤ *q* ≤ 64.
(5) The 64-bit binary hashing sequence *PH*′(*q*) is XORed with the watermarking extraction key Key(*i*, *j*) to extract the watermarking image *BW*′(*i*, *j*) from the medical image to be tested *F*′(*i*, *j*):
  (23) $$\begin{array}{c} BW^{\prime }\left(i,j\right)=PH^{\prime }\left(q\right)⊕\text{Key}\left(i,j\right), \end{array}$$
where 1 ≤ *q* ≤ 64, 1 ≤ *i* ≤ 64,1 ≤ *j* ≤ 64.
(6) The Hermite chaotic neural network is used to inversely scramble the extracted watermarking image *BW*′(*i*, *j*) to obtain the restored watermarking image *W*′(*i*, *j*).
(7) The normalized correlation coefficient (NC) is used to detect the restored watermarking image *W*′(*i*, *j*) obtained from the medical image to be tested *F*′(*i*, *j*). The normalized csrrelation coefficient formula is shown in the following formula:

(24) $$\begin{array}{c} \text{NC}=\frac{\sum_{i=1}^{M_{1}}\sum_{j=1}^{M_{2}}w\left(i,j\right)W^{\prime }\left(i,j\right)}{\sum_{i=1}^{M_{1}}\sum_{j=1}^{M_{2}}w\left(i,j\right)w\left(i,j\right)}. \end{array}$$

By comparing the NC value between the original watermarking image *W*(*i*, *j*) and the restored watermarking image *W*′(*i*, *j*), the restored watermarking image *W*′(*i*, *j*) obtained from the medical image to be tested is evaluated. The larger the NC value, the greater the correlation between the original watermarking image *W*(*i*, *j*) and the restored watermarking image *W*′(*i*, *j*).

The peak signal-to-noise ratio (PSNR) was used to evaluate the quality of the medical image to be tested. The PSNR formula is as follows:

(25) $$\begin{array}{c} \text{PSNR}=10\mathrm{lg}\frac{{\text{MAX}}^{2}}{\text{MSE}},\\ \text{MSE}=\frac{1}{N_{1}\times N_{2}}\sum_{i=1}^{N_{2}}\sum_{j=1}^{N_{1}}{\left[F\left(i,j\right)-F^{\prime }\left(i,j\right)\right]}^{2}, \end{array}$$

where MAX is the maximum gray value of the image pixel and MSE is the mean square error between the original image and the test image.

## 4. Experiment and Analysis

**Figure 9:**
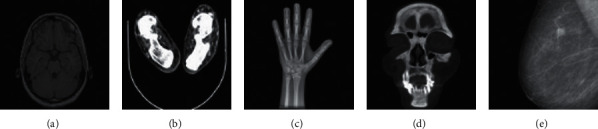
The original medical images.

**Figure 10:**
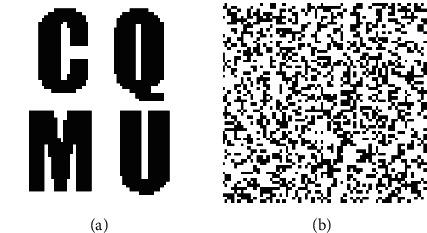
The watermarking images. (a) The original watermarking image. (b) The scrambled watermarking image.

In this work, several experiments are conducted by applying local nonlinear geometric attacks to investigate the performance and effectiveness of the proposed zero-watermarking algorithm. Five original medical images of different parts of the human body produced by different medical equipment are used to test the effectiveness of the proposed zero-watermarking algorithm. The original medical images with the size of 128 × 128 are shown in Figure 9. The original 64 × 64 watermarking image and the scrambled watermarking image are shown in Figure 10.

The following experiments test the ability of the watermarking algorithm to resist local nonlinear geometric attacks. For the convenience and repeatability of the experiment, we use the filter function of Adobe Photoshop software to implement the following common local distortion attacks. There are four types of local distortion attacks: ripple distortion attack, extrusion distortion attack, spherical distortion attack, and rotation distortion attack.

### 4.1. Ripple Distortion Attack

Ripple distortion attack is a common local nonlinear geometric attack, which transforms the coordinates of image pixels according to different functions.  Table 1 shows the experimental results under the ripple distortion attack when the distortion quantity increases from 150% to 750%.  Table 2 gives the restored watermarking images with the smallest NC value and their corresponding test medical image. The restored watermarking images can be clearly identified from Table 2, which shows that the proposed algorithm is very effective against ripple distortion attack.

### 4.2. Extrusion Distortion Attack

For extrusion distortion attack, distortion quantity is varied between 10% and 90% for simulation. The experimental results are shown in Table 3. All NC values are greater than 0.74. In Table 4, it is obvious that the restored watermarking image is very clear. The experimental results show that the proposed watermarking algorithm has good robustness against extrusion distortion attacks.

### 4.3. Spherical Distortion Attack

Then, we test the robustness of the proposed method on image spherical distortion. The test medical images were attacked by spherical distortions of 10%, 30%, 50%, 70%, and 90% in turn. Experimental results are listed in Table 5. It can be observed from Table 5 that most of the NC values are close to 1.0. The restored watermarking image in Table 6 further proves that the proposed algorithm has excellent performance under the spherical distortion attack.

### 4.4. Rotation Distortion Attack

We also evaluate the robustness of the proposed algorithm under rotation distortion attack.  Table 7 shows that when the distortion quantity increases, the NC value and the PSNR values gradually decrease. As can be seen from Table 8, even if the NC value is 0.5621, the watermark image can still be accurately extracted. These results demonstrate that the proposed algorithm has better robustness against the rotation distortion attack.

The above experimental results indicate that the proposed algorithm has strong capability against all the four types of local distortion attacks. Because it is a local nonlinear attack, the NC value does not necessarily decrease with the increase of the distortion quantity, and the change of the NC value is not the same as the change of the PSNR value.

## 5. Algorithm Comparison

In this section, the proposed algorithm is compared with two representative medical image watermarking algorithms. The details of these three algorithms are shown in Table 9. These three algorithms are robust medical image watermarking algorithms. Every robust watermarking algorithm can resist all kinds of attacks with good robustness and security and can solve all kinds of information security problems of the medical image. The proposed algorithm and [25, 26] are zero-watermarking algorithms. Their watermarking is based on the important features of the medical image rather than modifying the content of the medical image. They are easy to implement, fast to embed and extract, and have the advantages of small amount of computation and high speed. Therefore, the proposed algorithm and [25, 26] are very suitable for medical images. In these algorithms, DWT, DCT, and DFT are all linear transformations. However, VGG19 is a multilayer perceptron specially designed for image recognition. Its feature extraction is essentially a nonlinear transformation.

In order to further analyze the performance of the proposed algorithm, the algorithm [25, 26] has also carried out local nonlinear geometric attack experiments under the same conditions. Their results are compared with the experimental results of the proposed algorithm.

**Figure 11:**
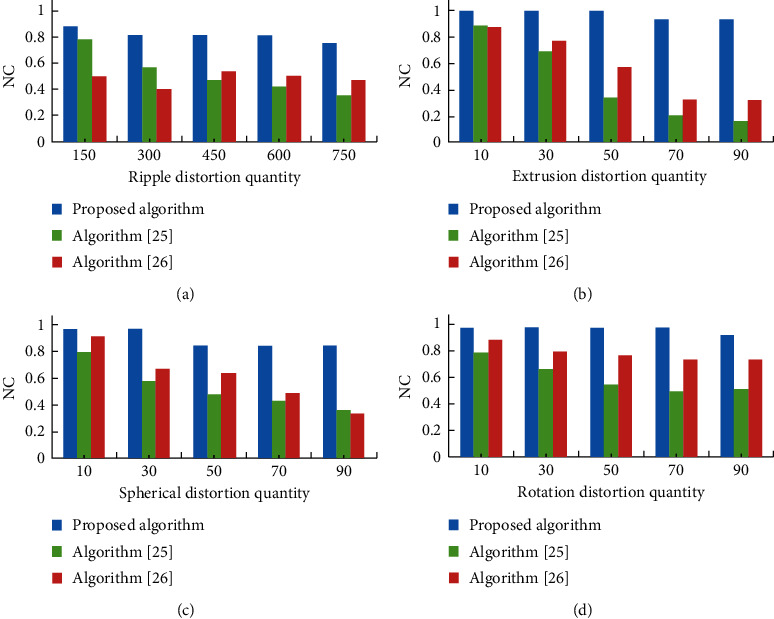
Robustness comparison between the proposed algorithm and two algorithms [25, 26] against four types of local nonlinear geometric attacks. (a) Ripple distortion attack. (b) Extrusion distortion attack. (c) Spherical distortion attack. (d) Rotation distortion attack.

The comparison of the algorithms under four types of local nonlinear geometric attacks is shown in Figure 11. As the blue bar of Figure 11(a) shows, it is obvious that the ability of the proposed algorithm to resist ripple distortion attack is better than the other two algorithms [25, 26]. The comparison of the experimental results of the extrusion distortion attack is shown in Figure 11(b). Figure 11(b) shows that, with the increase of extrusion distortion quantity, the NC value of the algorithm is close to 1, which is obviously higher than that of the other two algorithms [25, 26]. Especially when the extrusion distortion quantity is more than 70%, the difference of NC value is more significant.  Figure 11(c) shows the comparison under the spherical distortion attack. The NC values of the proposed algorithms are all greater than 0.87. This proves that the proposed algorithm is more resistant to the spherical distortion attack. It can be observed from Figure 11(d) that the three algorithms can effectively resist the rotation distortion attack, but the proposed algorithm has a stronger ability.

With the above comparison and analysis of the four local nonlinear geometric attacks, the proposed algorithm is obviously stronger than the other two algorithms in resisting local nonlinear geometric attacks. Therefore, the proposed algorithm has good robustness and can well resist local nonlinear geometric attacks.

## 6. Conclusions

In recent years, the medical image watermarking algorithm against geometric attacks has been a hot and difficult topic in the research of robust watermarking technology. In this paper, a zero-watermarking algorithm based on VGG19 is designed to resist local nonlinear geometric attacks. VGG19 is used to extract deep features of the medical image, and two-dimensional discrete Fourier transform and mean-perceptual hashing algorithm are used to generate zero-watermarking. The design process of the algorithm combines the concepts of deep convolution neural network, Fourier transform, perceptual hashing, cryptography, and zero-watermarking, which solves the problem of watermarking resisting local nonlinear geometric attacks. At the same time, the scrambling of the watermarking image ensures the security of the algorithm. It has high practical value for medical information protection.

## Figures and Tables

**Table 1:** Experimental results under ripple distortion attack.

	Distortion quantity (%)	150	300	450	600	750
*Medical image A*	PSNR (dB)	12.7889	11.9036	11.5765	11.2648	11.0596
	NC	0.87595	0.81021	0.81021	0.81106	0.74818

*Medical image B*	PSNR (dB)	16.6378	12.8092	12.8092	11.7588	11.0855
	NC	0.87595	0.87595	0.87595	0.68929	0.75075

*Medical image C*	PSNR (dB)	21.8267	17.8745	15.8378	14.6473	13.8828
	NC	0.81363	0.74989	0.81163	0.65842	0.59668

*Medical image D*	PSNR (dB)	21.3456	17.7586	16.1687	14.8927	14.0789
	NC	0.87509	0.87138	0.81021	0.81392	0.81392

*Medical image E*	PSNR (dB)	28.1822	25.2023	23.3165	21.9457	20.5296
	NC	0.87823	0.75104	0.68987	0.68872	0.68872

**Table 2:** The images under ripple distortion attack.

	Medical image A	Medical image B	Medical image C	Medical image D	Medical image E
The smallest NC value	0.74818	0.68929	0.59668	0.81021	0.68872
The restored watermarking image	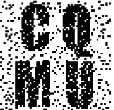	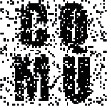	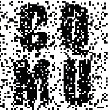	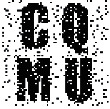	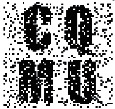
Medical image to be tested	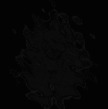	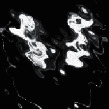	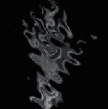	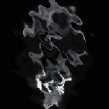	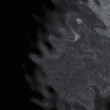

**Table 3:** Experimental results under extrusion distortion attack.

	Distortion quantity (%)	10	30	50	70	90
*Medical image A*	PSNR (dB)	13.0494	11.4873	10.6621	10.2615	9.9121
	NC	1.0	1.0	1.0	0.9354	0.9354

*Medical image B*	PSNR (dB)	21.7239	16.4862	14.1855	12.7081	11.6212
	NC	1.0	0.84222	0.7815	0.81049	0.81278

*Medical image C*	PSNR (dB)	26.561	19.8342	17.1758	15.695	14.9073
	NC	0.93826	0.93826	0.74818	0.74818	0.81163

*Medical image D*	PSNR (dB)	24.4511	18.0799	15.8822	14.692	14.0065
	NC	0.93626	0.81021	0.78076	0.78076	0.87395

*Medical image E*	PSNR (dB)	32.0185	26.229	23.5813	21.8296	20.504
	NC	0.9394	0.9394	0.9394	0.87566	0.87766

**Table 4:** The images under extrusion distortion attack.

	Medical image A	Medical image B	Medical image C	Medical image D	Medical image E
The smallest NC value	0.9354	0.7815	0.74818	0.78076	0.87566
The restored watermarking image	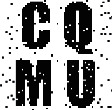	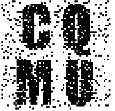	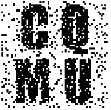	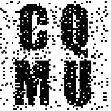	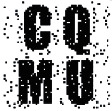
Medical image to be tested	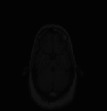	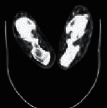	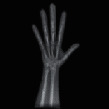	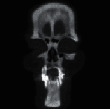	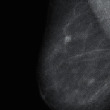

**Table 5:** Experimental results under spherical distortion attack.

	Distortion quantity (%)	10	30	50	70	90
*Medical image A*	PSNR (dB)	12.8585	11.4917	11.1459	11.3035	11.3435
	NC	1.0	1.0	0.87166	0.87166	0.87166

*Medical image B*	PSNR (dB)	18.4985	14.4427	13.0098	11.7524	10.5874
	NC	0.93654	0.87366	0.81221	0.81221	0.74704

*Medical image C*	PSNR (dB)	27.259	19.872	16.6028	14.5081	13.0453
	NC	0.93512	1.0	1.0	1.0	0.93997

*Medical image D*	PSNR (dB)	23.9151	17.1131	14.5852	13.2542	12.4597
	NC	1	1	1	1	0.93769

*Medical image E*	PSNR (dB)	30.64	25.4487	22.91	20.9156	19.2908
	NC	0.9394	1	1	0.81763	0.62984

**Table 6:** The images under spherical distortion attack.

	Medical image A	Medical image B	Medical image C	Medical image D	Medical image E
The smallest NC value	0.87166	0.74704	0.93512	0.93769	0.62984
The restored watermarking image	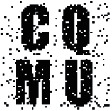	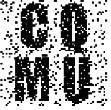	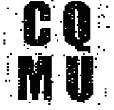	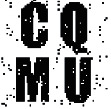	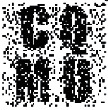
Medical image to be tested	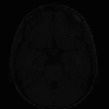	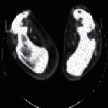	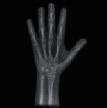	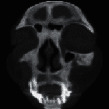	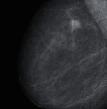

**Table 7:** Experimental results under rotation distortion attack.

	Distortion quantity (%)	10	30	50	70	90
*Medical image A*	PSNR (dB)	13.8738	13.2751	12.8138	12.5202	12.2938
	NC	1.0	1.0	1.0	1.0	0.94054

*Medical image B*	PSNR (dB)	21.5541	16.336	14.1869	12.7102	11.5936
	NC	0.93883	0.81049	0.87509	0.62698	0.62927

*Medical image C*	PSNR (dB)	23.2593	16.2611	14.4212	13.6166	13.1196
	NC	0.93626	0.93626	0.93626	0.93626	0.5621

*Medical image D*	PSNR (dB)	23.6471	17.9937	16.1955	15.1909	14.7219
	NC	0.94054	0.94054	0.90682	0.90682	0.90682

*Medical image E*	PSNR (dB)	32.9927	27.8346	25.4521	23.7641	22.4491
	NC	0.9394	0.9394	0.87823	0.87823	0.8182

**Table 8:** The images under rotation distortion attack.

	Medical image A	Medical image B	Medical image C	Medical image D	Medical image E
The smallest NC value	0.94054	0.62698	0.5621	0.90682	0.8182
The restored watermarking image	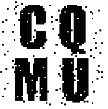	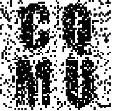	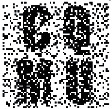	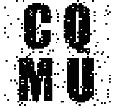	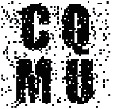
Medical image to be tested	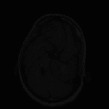	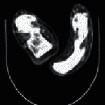	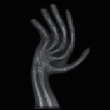	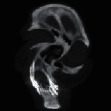	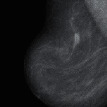

**Table 9:** Comparison between the proposed algorithm and other algorithms.

Algorithms	Objective	Method	Medical image content	Transform used
Algorithm [25]	Medical image security	Zero-watermarking	No change	DWT-DFT
Algorithm [26]	Medical image security	Zero-watermarking	No change	DCT
The proposed algorithm	Medical image security	Zero-watermarking	No change	VGG19-DFT

## Data Availability

The data used to support the findings of this study are available from the corresponding author upon reasonable request.
